# Real-world efficacy and safety of capecitabine with oxaliplatin in patients with advanced adenocarcinoma of the ampulla of Vater

**DOI:** 10.1186/s12885-024-12398-0

**Published:** 2024-05-23

**Authors:** Seunghwan Lee, Se Jun Park, Kabsoo Shin, Tae Ho Hong, In-Ho Kim, Myung Ah Lee

**Affiliations:** 1 Division of Medical Oncology, Department of Internal Medicine, Seoul St. Mary’s Hospital, College of Medicine, The Catholic University of Korea, 222 Banpo-daero, Secho-gu, Seoul, Korea; 2 Cancer Research Institute, College of Medicine, The Catholic University of Korea, Seoul, Korea; 3 Department of General Surgery, Seoul St. Mary’s Hospital, College of Medicine, The Catholic University of Korea, Seoul, Korea

**Keywords:** Ampulla of vater carcinoma, Capecitabine, Oxaliplatin, Chemotherapy, Treatment efficacy

## Abstract

**Background:**

Adenocarcinoma of the ampulla of Vater (AoV) is one of the rare periampullary cancers, and due to its anatomical location, it is categorized into various histologic subtypes. Its rarity and diversity pose challenges in treatment decision-making for patients with advanced AoV carcinoma. This study investigated the efficacy and safety of the combined regimen of capecitabine and oxaliplatin (CAPOX) in a real-world clinical setting.

**Methods:**

This investigation encompassed patients with advanced AoV carcinoma who underwent CAPOX treatment. Histologic phenotypes were identified through a combination of histopathological analysis and protein expression markers, including MUC1, CDX2, CK20, and MUC2. The correlation between histopathological determinants and survival outcomes was explored, in addition to an evaluation of the safety profile of CAPOX therapy.

**Results:**

From January 2010 to June 2023, 42 patients received CAPOX. Of these, 14 patients (33.3%) had not received any prior palliative chemotherapy, while 28 patients (66.7%) had undergone one prior line of chemotherapy. At a median follow up of 9.0 months, the median progression-free survival (PFS) was 4.38 months (95% CI, 2.78–5.69) and the median overall survival (OS) was 9.57 months (95% CI 7.56–11.6). The objective response and disease control rates were 38.1% and 61.9%, respectively. Patients who received CAPOX as a second-line treatment had poorer PFS (HR = 2.62; 95% CI, 1.49–4.90, *p* = 0.003) and OS (HR = 2.82, 95% CI, 1.47–5.38, *p* = 0.001) compared to those who received CAPOX as a first-line chemotherapy. There were no statistically significant differences in PFS (*p* = 0.185) and OS (*p* = 0.097) between groups based on histologic subtypes. Neutropenia (14.3%) emerged as the predominant grade 3–4 toxicity. Notably, treatment cessation occurred in select instances owing to grade 3 fatigue (9.5%) and peripheral neuropathy (9.5%).

**Conclusions:**

This study confirmed the therapeutic efficacy and safety of CAPOX in a real-world setting, consistent with prior phase II trial results. While CAPOX proved feasible for advanced AoV carcinoma regardless of histologic subtype, its reduced effectiveness in second-line settings necessitates further research to determine its optimal palliative use.

**Supplementary Information:**

The online version contains supplementary material available at 10.1186/s12885-024-12398-0.

## Background

The ampulla of Vater (AoV) carcinoma originates in the mucosa of the common channel where the pancreatic and common bile duct (CBD) converge. Given its anatomical location, distinguishing AoV carcinoma from malignancies in the pancreatic head, distal CBD, or duodenum can be challenging. As a form of periampullary cancer, AoV carcinoma is rare, comprising approximately 7% of all cases [1]. For patients with localized AoV carcinoma, surgical resection remains the primary curative treatment, yet the recurrence rate is high, with a reported 5-year survival rate of only 45% [2]. In patients with recurrent or metastatic disease, the prognosis significantly worsens, with a 2-year overall survival (OS) rate of just 5% [3]. 

For patients with advanced AoV carcinoma, gemcitabine and cisplatin have frequently been used as first-line palliative chemotherapy according to the ABC-02 clinical trial [4]. Moreover, the capecitabine and oxaliplatin (CAPOX) regimen has been suggested as another first-line palliative chemotherapy, given its meaningful effectiveness in a phase II trial for patients with adenocarcinoma of the small bowel and AoV [5]. Nonetheless, the strength of these findings is limited given that both studies were based on a small cohort of AoV carcinoma patients. For patients who progressed on gemcitabine-based chemotherapy, fluorouracil (5-FU) based treatments, such as 5-FU, leucovorin plus oxaliplatin (FOLFOX) or 5-FU, leucovorin plus irinotecan, could be considered [6, 7]. In the recently published ABC-06 trial, a phase III trial comparing FOLFOX as second-line treatment with best supportive care in patients with advanced biliary tract cancer, 11 cases of AoV carcinoma were included in the experimental arm. Compared to patients with biliary tract cancer, those with AoV carcinoma tended to show a greater survival benefit from FOLFOX [7]. However, given that these studies also included a limited number of patients with AoV carcinoma, the supporting evidence is less than compelling.

Based on histological features and specific immunohistochemical (IHC) markers, AoV carcinomas may be stratified into two histologic subtypes: Pancreato-biliary (PB) type and Intestinal type [8, 9]. Despite the lack of supporting data, the selection of chemotherapy may be influenced by the histologic subtype, with a preference for gemcitabine-based therapy for the PB subtype and 5-FU based regimen for the intestinal subtype [1, 6]. Although the study included a small number of patients, the CAPOX regimen demonstrated more favorable efficacy outcomes for the intestinal subtype compared to the PB subtype among patients with advanced AoV carcinoma [10]. Recently, rather than determining treatment based on the histologic subtype according to IHC, clinicians have been basing their therapeutic decisions on the basis of molecular profiling, since some features have been suggested to have a putative prognostic value [11]. 

In this retrospective analysis, the efficacy and safety of the CAPOX regimen were examined in patients with advanced AoV carcinoma. Alongside, the study also classified histological subtypes to discern potential variations in CAPOX’s effectiveness based on these classifications. This study evaluated the patient characteristics that corresponded with improved survival outcomes in those treated with CAPOX.

## Methods

### Patients

The clinicopathological data and treatment outcomes of patients diagnosed with adenocarcinoma of the AoV were analyzed. These patients had received CAPOX as a palliative treatment at Seoul St. Mary’s Hospital, Catholic University of Korea, between January 2010 and June 2023. Patients with histologically confirmed adenocarcinoma of the AoV were eligible for the study. Prior chemotherapy for metastatic disease was allowed.

### Treatment

CAPOX regimen consisted of intravenous oxaliplatin at 130mg/m^2^ on day 1 and capecitabine at 750mg/m^2^ orally, twice daily on day 1 through 14 of each 21-day cycle. Treatment courses were halted if patients experienced disease progression, unacceptable toxicity, or chose to refuse the treatment. Serial computed tomography scans were conducted at baseline and every 6 or 9 weeks until disease progression. Radiographic tumor response assessments were performed according to the Response Evaluation Criteria In Solid Tumors (RECIST) version 1.1. Serum carbohydrate antigen 19 − 9 (CA 19 − 9) was assessed at baseline and each tumor response evaluation. Adverse events were evaluated in accordance with the National Cancer Institute’s Common Terminology Criteria for Adverse Events, version 4.03. Physicians had the discretion to adjust chemotherapy dosages and schedules based on individual patient needs.

### Immunohistochemical assessment

To define the histologic phenotype of the AoV, IHC labeling on tissue microarray (TMA) samples was performed, assessing four key immunohistochemical markers: MUC1, CDX2, CK20, and MUC2. The process involved the deparaffinization of formalin-fixed, paraffin-embedded TMA sections using xylene, followed by a rehydration procedure. Subsequent immunostaining was carried out using an automated Bond-max immunostainer (Leica Microsystems, Newcastle, UK) after antigen retrieval. We used primary antibodies like anti-MUC1, anti-MUC2 (Novocastra, Newcastle, UK), anti-CDX2 (BioGenex, CA, USA), and anti-CK20 (Santa Cruz Biotechnology, TX, USA). The detection of antibody binding was facilitated by a Bond Polymer Refine Detection kit (Leica Microsystems, Vista, CA). Finally, samples were classified based on the IHC staining results, with consideration for cytoplasmic immunoreactivity for CK20 and MUC2, cell surface staining for MUC1, nuclear staining for CDX2, and using a modified H score for positive CDX2 expression. In instances of ambiguous staining results, the classification was based on the previously mentioned criteria guided by the expression of CDX2 and MUC1 [1, 9]. 

### Statistical analysis

Descriptive statistics are reported as proportions and medians, with ranges. The chi-square test or Fisher’s exact test was employed for the comparison of categorical variables, while the Student’s t-test was used for continuous variables. Progression-free survival (PFS) was defined as the time elapsed from the start of CAPOX to the date of disease progression as per RECIST version 1.1 or death, whichever occurred first. OS was estimated from the initiation of CAPOX to the last follow-up or any-cause death. Survival outcomes were evaluated using the Kaplan-Meier method and compared with the two-tailed log-rank test. To estimate the influence of clinicopathological factors on PFS and OS, multivariable regression was used based on the Cox proportional hazard model. A two-sided *p* value of less than 0.05 was deemed statistically significant. The statistical analyses were conducted using SPSS for Windows version 24.0 (IBM SPSS Inc., Armonk, New York, USA) and GraphPad Prism version 9.0 (GraphPad Software Inc., San Diego, CA, USA).

## Results

### Baseline characteristics

Between January 1, 2010, and June 30, 2023, 42 patients met the inclusion criteria for this study. The baseline demographic and clinical characteristics are outlined in Table 1. Approximately, one-quarter of the patients had well-differentiated histology, and three-quarters showed PB histologic subtype. Metastatic disease was present in 92.9% of the patients, and the majority of patients (88.1%) presented with recurrent disease. Among patients who underwent previous surgical resection, 16 patients (29.7%) were treated with 5-FU based adjuvant chemotherapy. Of the total, 14 patients (33.3%) did not receive any prior palliative chemotherapy, while 28 patients (66.7%) had experienced one prior line of palliative chemotherapy. Including those received adjuvant chemotherapy, 5-FU was previously administered as a component of either 5-FU/leucovorin or 5-FU/cisplatin regimen in 16 patients (38.1%). Metastasis was most identified in the liver (66.7%), followed by distant lymph nodes (47.6%), and the lung (40.5%). Elevated serum CA 19 − 9 levels were observed in 26 patients (61.9%) at baseline.

**Table 1:** Patient characteristics

Variables	CAPOX
	(*n* = 42)
**Age**, Median (Range)	62 (40–80)
<65 year, *n* (%)	25 (59.5)
≥65 year, *n* (%)	17 (40.5)
**Gender,** ***n*** **(%)**	
Male	24 (57.1)
Female	18 (42.9)
**Histologic grading,** ***n*** **(%)**	
Grade 1	10 (23.8)
Grade 2/3	32 (76.2)
**Histologic subtype,** ***n*** **(%)**	
Pancreato-biliary	31 (73.8)
Intestinal	11 (26.2)
**Disease status,** ***n*** **(%)**	
Locally advanced	3 (7.1)
Metastatic	39 (92.9)
**Prior tumor resection,** ***n*** **(%)**	
No (Initially advanced)	5 (11.9)
Yes (Recurrent disease)	37 (88.1)
Prior adjuvant chemotherapy*, ***n*** **(%)**	*n* = 37
No	26 (70.3)
Yes	16 (29.7)
**Prior lines of palliative chemotherapy,** ***n*** **(%)**	
0	14 (33.3)
1	28 (66.7)
**Prior first-line palliative chemotherapy,** ***n*** **(%)**	*n* = 28
Gemcitabine plus cisplatin	27 (96.4)
5-FU plus cisplatin	1 (3.6)
**Prior 5-FU containing chemotherapy,** ***n*** **(%)**	16 (38.1)
**Number of metastatic organ sites,** ***n*** **(%)**	
1–2	29 (69.0)
≥3	13 (31.0)
**Site of metastatic disease,** ***n*** **(%)**	
Liver	28 (66.7)
Lung	17 (40.5)
Peritoneum	7 (16.7)
Distant lymph node	20 (47.6)
**Baseline CA19-9 level,** ***n*** **(%)**	
Within normal (< 40U/mL)	16 (38.1)
Above normal (≥ 40U/mL)	26 (61.9)

CAPOX: capecitabine with oxaliplatin, 5-FU: fluorouracil, CA 19 − 9: carbohydrate antigen 19 − 9. *In a population with prior curative surgical resection

### Effectiveness outcomes

**Fig. 1:**
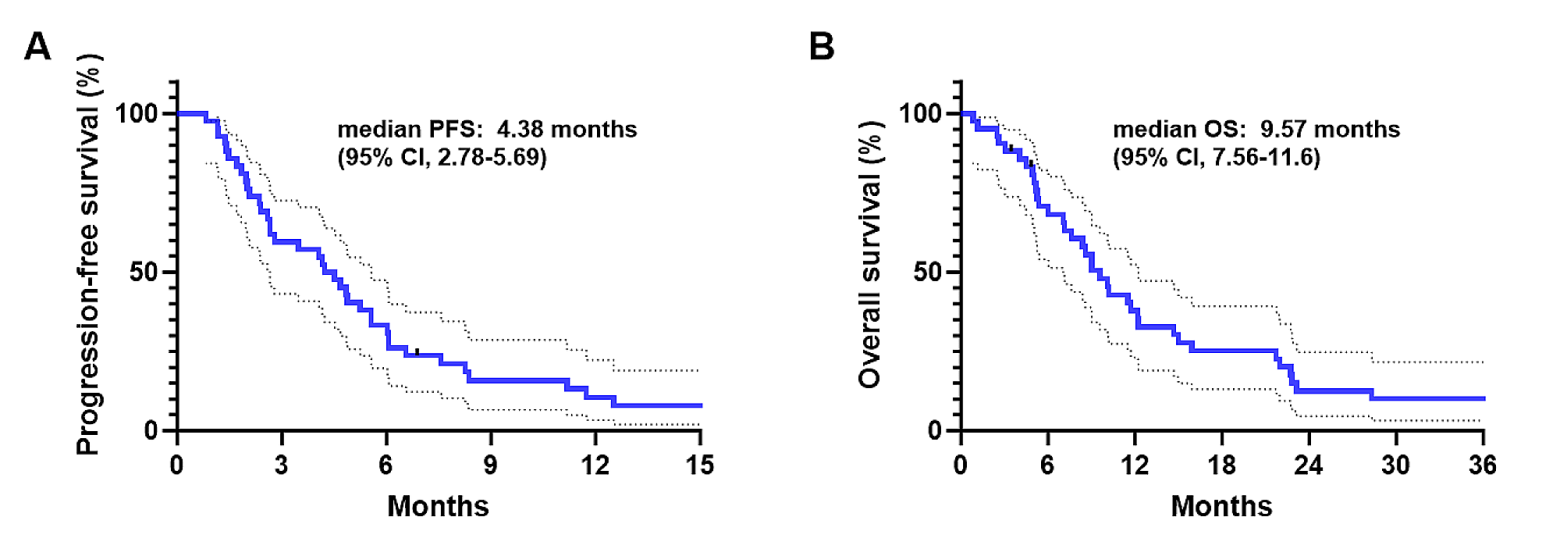
Kaplan-Meier estimates of progression-free survival (**A**) and overall survival (**B**) in patients who were treated with capecitabine and oxaliplatin for advanced ampulla of Vater cancer

Efficacy outcomes related to CAPOX are detailed in Table 2. The median follow-up duration was 9.0 months (95% CI, 6.03–12.2). Disease progression and death occurred in 39 patients (92.9%) and 37 patients (88.1%), respectively. The median PFS was 4.38 months (95% CI 2.78–5.69, Fig. 1A) and median OS was 9.57 months (95% CI 7.56–11.6, Fig. 1B). Six-month OS and PFS rates were 70.6% (95% CI 54.0-82.1) and 33.3% (95% CI 19.7–47.5), respectively. In terms of tumor response, partial response (PR) was achieved in 16 patients (38.1%), and stable disease (SD) was observed in 10 patients (23.8%), indicating a disease control rate of 61.9% with 6-month disease control rate standing at 38.1%. Among chemotherapy-naïve patients, 9 out of 14 (64.3%) showed a PR, and 3 (21.4%) exhibited SD with CAPOX as a first-line treatment. In the group that had previously received systemic chemotherapy, consisting of 28 patients, PR was achieved in 7 patients (25.0%), and SD was observed in another 7 patients (25.0%).

**Table 2:** Effectiveness outcomes

	CAPOX (*n* = 42)		
	Total	1st -line (*n* = 14)	2nd -line (*n* = 28)
**Best response,** ***n*** **(%)**			
PR	16 (38.1)	9 (64.3)	7 (25.0)
SD	10 (23.8)	3 (21.4)	7 (25.0)
PD	16 (38.1)	2 (14.3)	14 (50.0)
**Objective response rate,** ***n*** **(%)**	16 (38.1)	9 (64.3)	7 (25.0)
**Disease control rate,** ***n*** **(%)**	26 (61.9)	12 (85.7)	14 (50.0)
**6-month disease control rate,** ***n*** **(%)**	16 (38.1)	10 (71.4)	6 (21.4)
**Median PFS, months [95% CI]**	4.38 [2.78–5.69]	6.82 [4.28–7.85]	2.73 [0.74–4.57]
**6-month PFS rate, % [95% CI]**	33.3 [19.7–47.5]	71.4 [40.6–88.2]	14.3 [4.5–29.5]
**Median OS, months [95% CI]**	9.57 [7.56–11.6]	19.3 [1.11–30.7]	8.56 [5.52–11.6]
**6-month OS rate, % [95% CI]**	70.6 [54.0–82.1]	64.3 [34.3–83.3]	23.6 [9.7–41.0]

CAPOX: capecitabine with oxaliplatin, PR: partial response, SD: stable disease, PD: progressive disease, PFS: progression-free survival, CI: confidence interval, OS: overall survival

### Survival outcomes by prior chemotherapy

**Fig. 2:**
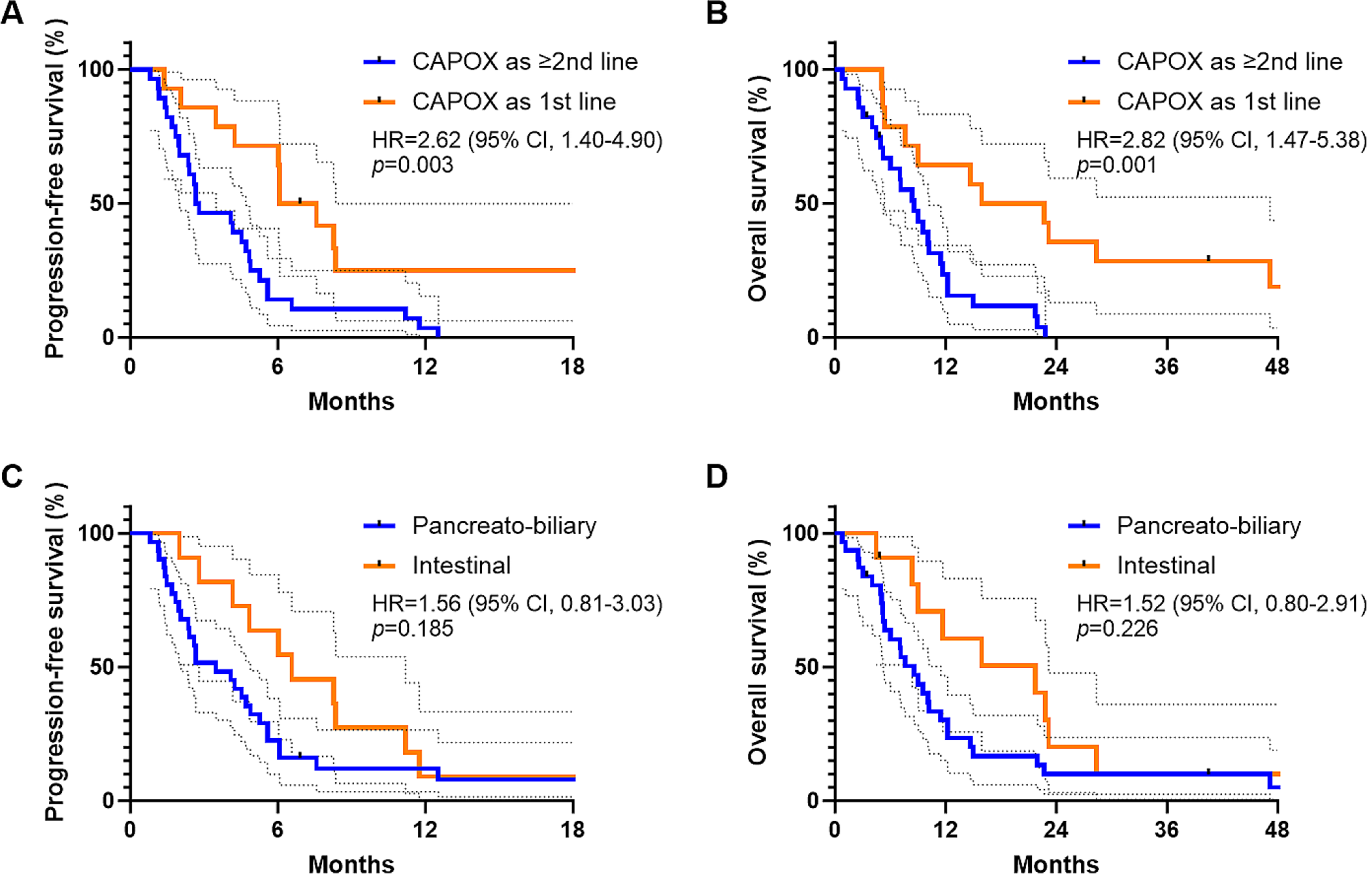
Survival outcome with capecitabine plus oxaliplatin according to the line of treatment and histologic subtypes. Progression-free survival (**A**) and overall survival (**B**) are stratified by line of systemic chemotherapy. Progression-free survival (**C**) and overall survival (**D**) are analyzed according to the histologic subtypes

The survival outcomes associated with CAPOX were analyzed with regard to whether patients had received prior palliative chemotherapy. Progressive disease was confirmed in all 28 patients who had received prior palliative chemotherapy, while 11 of 14 (78.6%) of those received CAPOX as a first-line treatment showed progressive disease. In patients subjected to prior palliative chemotherapy, the median PFS was 2.73 months (95% CI, 0.74–4.57), contrasting with 6.82 months (95% CI, 4.28–7.85) in those undergoing CAPOX as a first-line treatment (HR = 2.62; 95% CI, 1.40–4.90; *p* = 0.003; Fig. 2A). The 6-month PFS rate was 14.3% (95% CI, 4.5-29.5%) in patients who had received previous chemotherapy, compared to 71.4% (95% CI, 40.6-88.2%) in patients without any prior palliative chemotherapy. Death events were reported in 26 out of the 28 patients (92.9%) and 11 out of the 14 patients (78.6%) from these groups, respectively. The median OS was 8.56 months (95%CI, 5.52–11.6) in patients who had received previous chemotherapy, compared to 19.3 months (95% CI, 1.11–30.7) in patients who had not received any palliative chemotherapy (HR = 2.82; 95% CI, 1.47–5.38; *p* = 0.001; Fig. 2B). Corresponding one-year OS rates were 23.6% (95% CI, 9.7-41.0%) and 64.3% (95% CI, 34.3-83.3%), respectively.

### Survival outcomes by histologic subtypes

Within the context of survival outcomes differentiated by histological subtype, progressive disease was confirmed in 28 of the 31 patients (90.3%) with the PB subtype and in all 11 patients with the intestinal subtype. The PB group had a median PFS of 3.48 months (95% CI, 1.69–5.26) compared to 6.56 months (95% CI, 2.88–10.2) in the intestinal group (HR = 1.56; 95% CI, 0.81–3.01; *p* = 0.185; Fig. 2C). The 6-month PFS rate was noted at 22.6% (95% CI, 10.0-38.3%) in the PB subtype, versus 63.6% (95% CI, 29.7-87.5%) in the intestinal subtype. Death events accounted for 29 of 31 patients (93.5%) in the PB subtype and 9 of 11 patients (81.8%) in the intestinal subtype. The median OS was 8.56 months (95% CI, 5.94–11.2) in the PB group, compared to 21.7 months (95% CI, 6.61–36.9) in the intestinal group. While the PB subtype seemed to be associated with a shorter OS, the difference was not statistically significant (HR = 1.77; 95% CI, 0.90–3.49; *p* = 0.097; Fig. 2D). The one-year OS rate was found to be 30.2% (95% CI, 15.1-46.8%) in patients with the PB subtype, as opposed to 60.6% (95% CI, 25.8-83.1%) in patients with the intestinal subtype.

### Multivariate analysis of prognostic factors

Tables 3 and 4 present the multivariate analysis of survival outcomes, with subgroups categorized according to various clinicopathological factors. Patients who received CAPOX as a second line of treatment exhibited shorter PFS (HR = 2.31; 95% CI, 1.08–4.93; *p* = 0.031) and OS (HR = 3.16; 95% CI, 1.30–7.66; *p* = 0.011) compared to those treated with CAPOX as a first-line palliative chemotherapy. Furthermore, a poorer PFS was observed in patients over 70 compared to those under 70 years of age (HR = 2.72; 95% CI, 1.19–6.23; *p* = 0.018), whereas the discrepancy in OS between these age groups was not statistically significant (HR = 1.57; 95% CI, 0.67–3.67; *p* = 0.295). The univariate analysis identified an inferior PFS in patients who had been treated with 5-FU containing prior treatment (HR = 1.93; 95% CI, 0.96–3.87; *p* = 0.033) and in patients with liver metastases (HR = 1.97; 95% CI, 1.05–3.72; *p* = 0.048), however, these disparities did not present in the multivariate analysis.

**Table 3:** Univariate and multivariate evaluations of clinicopathologic factors in determining progression-free survival

	PFS			
Variables	Univariate analysis		Multivariate analysis	
	HR (95% CI)	*p* value	HR (95% CI)	*p* value
**Age ≥ 70 (vs. <70 year)**	**2.21 (0.88–5.54)**	**0.021**	**2.72 (1.19–6.23)**	**0.018**
**Prior palliative chemotherapy (vs. none)**	**2.62 (1.40–4.90)**	**0.003**	**2.31 (1.08–4.93)**	**0.031**
Histologic grade 2–3 (vs. grade 1)	2.04 (1.05–3.95)	0.060		
Pancreato-biliary type (vs. intestinal)	1.56 (0.81–3.03)	0.185		
Elevated CA19-9 level (vs. normal)	1.91 (1.00–3.65)	0.050		
**Prior 5-FU containing treatment (vs. none)**	**1.93 (0.96–3.87)**	**0.033**	**1.64 (0.84–3.19)**	**0.145**
**Liver metastases (vs. none)**	**1.97 (1.05–3.72)**	**0.048**	**2.31 (0.92–4.21)**	**0.083**
Lung metastases (vs. none)	1.09 (0.58–2.07)	0.779		
Number of organ metastases ≥ 3 (vs. 1–2)	1.22 (0.61–2.44)	0.551		

PFS: progression-free survival, HR: hazard ratio, CA 19 − 9: carbohydrate antigen 19 − 9, 5-FU: fluorouracil. Statistically significant variables are in bold font

**Table 4:** Univariate and multivariate analysis of clinicopathologic factors influencing overall survival

	OS			
Variables	Univariate analysis		Multivariate analysis	
	HR (95% CI)	*p* value	HR (95% CI)	*p* value
Age ≥ 70 (vs. <70 year)	1.92 (0.77–4.81)	0.074	1.57 (0.67–3.67)	0.295
**Prior palliative chemotherapy (vs. none)**	**2.82 (1.47–5.38)**	**0.001**	**3.16 (1.30–7.66)**	**0.011**
Histologic grade 2–3 (vs. grade 1)	1.73 (0.87–3.45)	0.160		
Pancreato-biliary type (vs. intestinal)	1.52 (0.80–2.91)	0.226		
Elevated CA19-9 level (vs. normal)	1.82 (0.95–3.46)	0.064		
Prior 5-FU containing treatment (vs. none)	1.59 (0.77–3.28)	0.208	1.30 (0.64–2.63)	0.472
Liver metastases (vs. none)	1.67 (0.87–3.19)	0.136	1.40 (0.66–2.96)	0.384
Lung metastases (vs. none)	1.61 (0.81–3.22)	0.131		
Number of organ metastases ≥ 3 (vs. 1–2)	1.58 (0.75–3.31)	0.230		

OS: overall survival, HR: hazard ratio, CA 19 − 9: carbohydrate antigen 19 − 9, 5-FU: fluorouracil. Statistically significant variables are in bold font

### Safety

The treatment-related toxicity profiles are summarized in Table 5. There were no treatment-related adverse events leading to death. Almost all patients (*n* = 38, 90.5%) experienced adverse events of any grade, while grade 3 or 4 adverse events were observed in 16 patients (38.1%). The most common treatment-related adverse events in patients receiving CAPOX were anemia (*n* = 19, 45.2%), fatigue (*n* = 17, 40.5%), neutropenia (*n* = 13, 31.0%), thrombocytopenia (*n* = 12, 28.6%), peripheral neuropathy (*n* = 9, 21.4%), and nausea (*n* = 9, 21.4%). Among these, neutropenia was the most common grade 3 or 4 adverse event (*n* = 6, 14.3%). Grade 3 or 4 peripheral neuropathy and fatigue were recorded in 4 patients (9.5%), each leading to permanent treatment discontinuation. Additionally, peripheral neurotoxicity, regardless of grade, led to the halt of treatment in 6 patients (14.3%). Detailed information regarding the duration of CAPOX therapy and the cumulative dosage of oxaliplatin administered to these subjects is compiled in supplementary Table 1.

**Table 5:** Treatment-related adverse events

Toxicity type (*n* = 42)	Any grade	Grade 3–4
All, n (%)	38 (90.5)	16 (38.1)
Neutropenia, *n* (%)	13 (31.0)	6 (14.3)
Anemia, *n* (%)	19 (45.2)	0
Thrombocytopenia, *n* (%)	12 (28.6)	1 (2.4)
Fatigue, *n* (%)	17 (40.5)	4 (9.5)
Peripheral neuropathy, *n* (%)	9 (21.4)	4 (9.5)
Hand-foot syndrome	7 (16.7)	0
Stomatitis, *n* (%)	5 (11.9)	1 (2.4)
Nausea, *n* (%)	9 (21.4)	3
Vomiting, *n* (%)	5 (11.9)	1 (2.4)
Diarrhea, *n* (%)	2 (4.8)	0

## Discussion

Given the rarity of this disease, our study assessed the survival outcomes in a relatively large cohort of patients with AoV carcinoma, who were treated with the CAPOX regimen. Additionally, this study evaluated the efficacy of the CAPOX regimen across different histological subtypes, using a widely accepted histological classification.

No prospective studies have evaluated the benefits of specific chemotherapy regimens in a large cohort of patients with advanced AoV carcinoma. Consequently, palliative chemotherapy for advanced AoV carcinoma has relied on limited published data, including various trials such as basket trials, which also permit the inclusion of ampullary cancer along with small intestinal or biliary duct cancers [4, 5]. In a prior prospective phase II study involving patients naïve to palliative chemotherapy, the response rate to the CAPOX regimen was established at 50%, with a median OS of 20.4 months [5]. Subgroup analysis from the same study suggested a reduced response rate for AoV carcinoma (*n* = 12) compared to small bowel adenocarcinoma (*n* = 18) (33% vs. 61%, respectively). The present analysis of 14 patients who received first-line treatment with CAPOX—yielding a response rate of 64.3% and a median OS of 19.3 months—suggests outcomes that compare well with previous findings.

The inferior efficacy of CAPOX in AoV carcinoma, as compared to small intestine adenocarcinoma may attributed to the complex histology of ampullary carcinomas, characterized by the confluence of duodenal, pancreatic, and biliary epithelium within the AoV [5]. A previous investigation, albeit limited by a small sample size, demonstrated a superior PFS in the intestinal subtype compared to the PB subtype in CAPOX therapy [10]. However, our study, which included a larger patient cohort and provided a more detailed delineation of histological subtypes, failed to identify any disparity in the efficacy of CAPOX according to histological subtype. It is possible that a subset of patients with AoV carcinoma harbor molecular alterations that serve as negative predictors of survival, potentially rendering them less responsive to CAPOX [12]. Nevertheless, in terms of prognosis, our findings are consistent with existing literature, indicating a trend toward poorer outcomes in patients with the PB subtype, compared to those with the intestinal subtype [9, 13]. 

In an analysis examining the correlation between prior 5-FU exposure and the efficacy of CAPOX treatment, the group that had previously received 5-FU-containing treatment tended to have a shorter PFS compared to the group that had not. However, no significant difference in PFS was observed when comparing patients who experienced disease progression within 6 months of 5-FU-containing chemotherapy (*n* = 7) and those whose disease progressed after 6 months (*n* = 9). Therefore, the relationship between the resistance to 5-FU and the efficacy of CAPOX treatment may not be clearly defined.

The safety profile of CAPOX reported in this real-world study aligns with the results of a prior phase II trial conducted among Western populations [5]. The hematological toxicity profile of the CAPOX regimen in this cohort was largely manageable, with only 14.3% of patients presenting with grade 3 or 4 neutropenia, and an absence of febrile neutropenia. Nonetheless, a notable challenge arose due to peripheral neurotoxicity, leading to the termination of treatment in a subset of patients (14.3%). This subgroup underwent a median of 6 treatment cycles (range, 6–13) and received a mean cumulative oxaliplatin dose of 564 mg/m² (standard deviation, ± 330 mg/m²). Given that oxaliplatin-induced chronic neurotoxicity is cumulative and dose-related, it poses a challenge in sustaining CAPOX therapy in certain patients [14, 15]. The cessation of oxaliplatin could be considered after 4 months of treatment while maintaining the administration of oral capecitabine until time of disease progression. The reduced capecitabine dose in the CAPOX regimen, as compared to its use in colorectal cancer treatment, was associated with a lower incidence of grade 3 hand-foot syndrome (0% vs. 6%) [16]. 

Commonly, the gemcitabine with cisplatin has employed as the principal therapeutic approach for patients with advanced AoV carcinoma. However, in the underlying clinical trial, the inclusion of patients with AoV carcinoma was notably sparse, constituting a mere 6.5% (*n* = 20) of the overall study cohort [4]. This limited proportion of patients with AoV carcinoma imposes a significant challenge in establishing a treatment guideline. Regardless of the histologic subtype, CAPOX demonstrated favorable efficacy outcomes when used as a first-line treatment in patients with advanced AoV carcinoma, making it a suitable first-line treatment option. Additionally, in the ABC-06 trial, patients with AoV carcinoma showed better outcomes with FOLFOX treatment compared to those with biliary tract cancer, suggesting that in selecting a palliative first-line regimen for AoV carcinoma patients, choosing treatment regimens based on histologic subtype, such as opting for CAPOX as a first-line treatment in cases of the intestinal subtype, could also be worth considering. However, considering the occurrence of permanent discontinuation due to grade 3 or higher fatigue and peripheral neurotoxicity, this regimen should be considered in patients with a relatively good performance status. Additionally, a response rate approaching 40% suggests that this regimen might be suitable for patients requiring rapid alleviation from tumor-associated symptoms. It also implies the potential of its use as adjuvant therapeutic approach for patients with resected localized ampullary cancer.

In second-line settings, CAPOX demonstrated only modest efficacy, suggesting that alternative treatment strategies might be warranted for such patients. Notably, while there was no significant disparity in therapeutic outcomes associated with histologic subtypes for the CAPOX regimen, the PB subtype manifested inferior PFS and OS outcomes. Given this backdrop, for patients with the PB subtype, alternative treatment strategies, such as 5-FU, leucovorin, and liposomal irinotecan, might be worth considering, given their demonstrated efficacy in biliary tract cancer patients [17]. Moreover, evaluating molecular alterations in AoV carcinoma patients is crucial to ascertain the potential for targeted treatments. This includes screening for potential therapeutically actionable genetic changes, such as mutations in the Kirsten rat sarcoma viral oncogene (*KRAS*)—notably the *KRAS* genotype G12C, *BRCA* 1/2 mutations, and gene mutations associated with DNA mismatch repair [18]. Additionally, in line with therapeutic approaches in colon cancer, the integration of CAPOX with antiangiogenic agents, based on *RAS* mutation status, merits thoughtful consideration.

There are some limitations to our study. First, this study was conducted retrospectively at a single institution. Secondly, in the context of relapsed patients with initially ambiguous phenotypes, we were unable to identify a dominant subtype due to the absence of repeat biopsies post-recurrence. Third, certain patients presented with oligometastatic disease, and local treatments administered to these patients might have influenced survival outcomes. Lastly, the limited sample size hampers comparisons between various patient subgroups within the study.

## Conclusions

In the present study, the therapeutic efficacy and safety of CAPOX were corroborated in a real-world clinical environment, aligning with outcomes reported in a prior phase II trial. This study demonstrated the efficacy and safety of CAPOX in a real-world clinical setting, and its effect in the first-line setting showed results consistent with those of a previous phase II trial. No difference in the effectiveness of CAPOX according to histologic subtype was observed, indicating that CAPOX can be a feasible treatment option for patients with advanced AoV carcinoma irrespective of the subtype. Nevertheless, with its limited impact observed in the second-line settings and beyond, further investigations are essential to discern the optimal patient cohorts for CAPOX as a palliative chemotherapeutic approach.

### Electronic supplementary material

Below is the link to the electronic supplementary material.

Supplementary Material 1

## Data Availability

The datasets used in the current study are available from the corresponding author on request.

## Abbreviations

AoV: ampulla of Vater
CBD: common bile duct
OS: overall survival
CAPOX: capecitabine and oxaliplatin
5-FU: fluorouracil
FOLFOX: fluorouracil, leucovorin plus oxaliplatin
IHC: immunohistochemistry
PB: Pancreato-biliary
RECIST: Response Evaluation Criteria In Solid Tumors
CA 19 − 9: carbohydrate antigen 19 − 9
TMA: tissue microarray
PFS: progression-free survival
PR: partial response
SD: stable disease
KRAS: Kirsten rat sarcoma viral oncogene
