# Supplementary material for: Real-world efficacy and safety of capecitabine with oxaliplatin in patients with advanced adenocarcinoma of the ampulla of Vater
Source: BMC Cancer. 2024 May 23;24:634. doi: 10.1186/s12885-024-12398-0 (PMC11119299; doi:10.1186/s12885-024-12398-0)
Supplement: Supplementary file 1 — Supplementary Material 1 [file 12885_2024_12398_MOESM1_ESM.docx]

**Table S1. Dose intensity in patients who discontinued treatment permanently due to peripheral neurotoxicity**

|  | **CAPOX (n=6)** | |
| --- | --- | --- |
| **Median duration of treatment, months (range)** | | **5.7 (3.5-21.3)** |
| **Median cycles of treatment, n (range)** | | **6 (6-13)** |
| **Cumulative oxaliplatin dose, mg/m^2^**  **Mean (SD)** | | **564 (330)** |

*CAPOX* capecitabine with oxaliplatin, *SD* standard deviation
